# A Compact, Multifunctional Fusion Module Directs Cholesterol-Dependent Homomultimerization and Syncytiogenic Efficiency of Reovirus p10 FAST Proteins

**DOI:** 10.1371/journal.ppat.1004023

**Published:** 2014-03-20

**Authors:** Tim Key, Roy Duncan

**Affiliations:** 1 Department of Microbiology and Immunology, Dalhousie University, Halifax, Nova Scotia, Canada; 2 Department of Biochemistry and Molecular Biology, Dalhousie University, Halifax, Nova Scotia, Canada; 3 Department of Pediatrics, Dalhousie University, Halifax, Nova Scotia, Canada; Georgia State University, United States of America

## Abstract

The homologous p10 fusion-associated small transmembrane (FAST) proteins of the avian (ARV) and Nelson Bay (NBV) reoviruses are the smallest known viral membrane fusion proteins, and are virulence determinants of the fusogenic reoviruses. The small size of FAST proteins is incompatible with the paradigmatic membrane fusion pathway proposed for enveloped viral fusion proteins. Understanding how these diminutive viral fusogens mediate the complex process of membrane fusion is therefore of considerable interest, from both the pathogenesis and mechanism-of-action perspectives. Using chimeric ARV/NBV p10 constructs, the 36–40-residue ectodomain was identified as the major determinant of the differing fusion efficiencies of these homologous p10 proteins. Extensive mutagenic analysis determined the ectodomain comprises two distinct, essential functional motifs. Syncytiogenesis assays, thiol-specific surface biotinylation, and liposome lipid mixing assays identified an ∼25-residue, N-terminal motif that dictates formation of a cystine loop fusion peptide in both ARV and NBV p10. Surface immunofluorescence staining, FRET analysis and cholesterol depletion/repletion studies determined the cystine loop motif is connected through a two-residue linker to a 13-residue membrane-proximal ectodomain region (MPER). The MPER constitutes a second, independent motif governing reversible, cholesterol-dependent assembly of p10 multimers in the plasma membrane. Results further indicate that: (1) ARV and NBV homomultimers segregate to distinct, cholesterol-dependent microdomains in the plasma membrane; (2) p10 homomultimerization and cholesterol-dependent microdomain localization are co-dependent; and (3) the four juxtamembrane MPER residues present in the multimerization motif dictate species-specific microdomain association and homomultimerization. The p10 ectodomain therefore constitutes a remarkably compact, multifunctional fusion module that directs syncytiogenic efficiency and species-specific assembly of p10 homomultimers into cholesterol-dependent fusion platforms in the plasma membrane.

## Author Summary

Natural infections by fusogenic orthoreoviruses can result in severe afflictions ranging from neuropathogenicity to pneumonia and death. The fusogenic capacity of these viruses, attributable to a unique family of fusion-associated small transmembrane (FAST) proteins, is a correlate of virulence. The FAST proteins are the only known examples of nonenveloped virus membrane fusion proteins, and they are the smallest known viral fusogens whose structural and functional attributes are incompatible with current models of protein-mediated membrane fusion. Exploiting the sequence divergence and distinct syncytiogenic rates of representative p10 FAST proteins from avian and bat reovirus isolates, we determined the p10 ectodomain is a compact, complex fusion module comprising two independent functional motifs. One motif determines species-specific p10 fusion efficiency by governing formation of a cystine loop fusion peptide, while the other directs reversible clustering and multimerization of p10 in cholesterol-dependent membrane microdomains. Remarkably, a juxtamembrane tetra-peptide is solely responsible for co-dependent clustering and multimerization of p10 in distinct, species-specific fusion platforms. This is the first example of a viral fusogen utilizing a membrane-proximal ectodomain region (MPER) to direct cholesterol-dependent multimerization and assembly into fusion platforms.

## Introduction

More than two new species of human-infecting viruses are reported worldwide each year, and these novel viruses pose a substantial threat of becoming emerging human infections [1]. Bat populations may serve as a major reservoir for emerging human viral infections [2]–[5]. Genus *Orthoreovirus* is one of 15 recognized genera in Family *Reoviridae*, a large, diverse group of non-enveloped viruses with segmented, double-stranded RNA genomes [6]. Several novel orthoreoviruses of pteropine origin have recently been isolated, and six of these isolates were associated with acute respiratory infections in humans [7]–[11]. Moreover, a serological survey revealed 13% of 109 random serum samples of subjects in Malaysia were seropositive for pteropine reoviruses [12], indicating zoonotic transmission of fusogenic bat reoviruses may be a frequent event. Monitoring agencies have recommended close monitoring of orthoreovirus evolution, as well as a necessity for further research to allow for prevention and control measures to be taken in the event of zoonotic transmission [13].

Genus *Orthoreovirus* is divided into two subgroups, fusogenic and non-fusogenic orthoreoviruses, based on the ability to induce cell-cell fusion and syncytium formation [14]. Human infections with non-fusogenic mammalian reoviruses (MRVs) are commonplace and generally asymptomatic [15]. In contrast, natural infections with fusogenic orthoreoviruses induce an array of pathologies in virus-infected animals; baboon reovirus (BRV) is associated with meningoencephalomyelitis in juvenile baboons [16], reptilian reoviruses (RRVs) cause pneumonia and neurological dysfunction [17], [18], and avian reoviruses (ARVs) are the causative agents of a wide range of diseases that include arthritis and enteric disease syndromes [19]. Syncytium formation is mediated by non-structural, fusion-associated small transmembrane (FAST) proteins encoded by all fusogenic orthoreoviruses, and by fusogenic aquareoviruses in the closely related Genus *Aquareovirus* [20]. These are the only known membrane fusion proteins encoded by non-enveloped viruses. The FAST proteins are not involved in viral entry, but instead induce fusion between virus-infected and neighboring uninfected cells, thereby promoting virus dissemination [21]. The extent of syncytiogenesis correlates with viral pathogenicity [22], and mice intranasally infected with a chimeric, FAST protein-expressing vesicular stomatitis virus (VSV) showed increased neuropathology [23]. The FAST proteins are therefore virulence determinants of the fusogenic reoviruses.

There are six current members of the FAST protein family, each named according to their molecular mass: the orthoreovirus FAST proteins, p10, p13, p14 and p15 are encoded by ARV and Nelson Bay reovirus (NBV), Broome reovirus (BroV), RRV, and BRV, respectively [24]–[27], while fusogenic aquareoviruses encode p16 and p22 FAST proteins [28], [29]. Recent phylogenetic analysis suggests the fusogenic reoviruses arose from an ancestral, non-fusogenic virus by at least two separate gain-of-function events, possibly involving divergent evolution from one or more precursor non-structural proteins that were membrane-interactive, but non-fusogenic, [20], [30]. The FAST proteins share no identifiable sequence similarity with each other, or with any other known membrane fusion protein, and each possesses a unique repertoire and arrangement of functional motifs. Defining attributes of family members include their small size (95–198 amino acids), presence of a single-pass transmembrane domain (TMD) with N_exoplasmic_/C_endoplasmic_ membrane topology resulting in ectodomains of <40 residues, sites for fatty-acid modification (N-terminal myristoylation or palmitoylation of membrane proximal Cys residues), a cluster of membrane-proximal basic residues on the cytoplasmic side of the TMD, a short hydrophobic or amphipathic motif that can be located on either side of the TMD, and an intrinsically disordered cytoplasmic tail [25], [27], [31]–[33]. Determining how these motifs function in a coordinated manner to mediate membrane fusion is critical to our understanding of how these unusual viral fusion proteins mediate syncytiogenesis, and how this process influences viral pathogenesis.

Studies of enveloped viral fusion proteins suggest that all membrane fusion reactions share a common pathway progressing through membrane binding, close membrane apposition, outer-leaflet mixing, and pore formation/expansion [34]. Although enveloped virus fusion proteins possess considerable architectural diversity, remarkable similarities exist in the dynamic structural rearrangements undertaken throughout the fusion reaction [35]. Triggered conformational changes result in extensive structural remodeling of complex, multimeric ectodomains that expose and project hydrophobic fusion peptides (FPs) toward the target cell membrane, followed by folding back of the extended intermediate structure into a compact trimeric hairpin structure. FPs are critical for fusion activity, they are extremely sensitive to mutation, and can exist as either internal fusion loops or as N-terminal α-helices [36]. Membrane merger is believed to be mediated by bilayer deformation, using the energy released as the metastable, pre-fusion structure transitions to the lowest energy, post-fusion conformation and/or by membrane curvature effects induced by FP partitioning into bilayers [34], [37]. Membrane-proximal external regions (MPERs) of several enveloped viral fusion proteins have also been shown to play active roles in the membrane fusion reaction [38]–[41]. These short, hydrophobic, frequently tryptophan-rich sequences partition into lipid bilayers where they may influence bilayer stabilization or curvature [42]. Despite extensive study of numerous enveloped virus fusogens, a clear picture on the specific role of FPs, MPERS, and quaternary structural transitions in the fusion reaction has not emerged.

**Figure 1:**
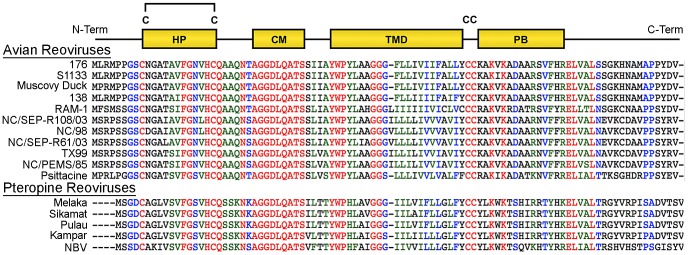
Sequence and motif conservation in avian and pteropine reovirus p10 FAST proteins. Sequences of representative isolates of avian and pteropine p10 clades are shown, color coded to indicate the degree of conservation: red, absolutely conserved; blue, highly conserved; green, moderately conserved; black, not conserved. The diagram at the top depicts locations of motifs present in the ectodomain (HP, hydrophobic patch; CM, conserved motif) and endodomain (PB, polybasic) flanking the central transmembrane domain (TMD). The four conserved cysteine residues (C) are shown; the di-cysteine motif in the endodomain is palmitoylated while the two cysteines in the ectodomain form an intra-molecular di-sulfide bond.

At 95–98 residues, the p10 FAST proteins of ARV and NBV are the smallest known protein fusogens and provide a simple system for investigating the mechanism of protein-mediated membrane fusion. As nonstructural proteins involved in cell-cell fusion, not virus-cell fusion, the relationship between syncytium formation and viral pathogenesis can also be analyzed in the absence of confounding virus entry effects. The p10 proteins are the only FAST proteins with numerous homologs, which segregate into two host-specific clades (Figure 1); an avian clade and the newly emerged pteropine reoviruses associated with severe human respiratory infections. The rate of syncytiogenesis varies considerably between representatives of the avian and pteropine clades [21]. There is extensive sequence divergence between p10 clades (<30% amino acid identity), but they share an identical arrangement of structural motifs (Figure 1). The ARV and NBV p10 FAST proteins comprise a central transmembrane domain flanked by approximately equal sized (∼40 residue) ecto- and endodomains (Figure 1). The ectodomain contains a moderately hydrophobic region, termed the hydrophobic patch (HP), flanked by two conserved Cys residues, which were shown to form an intramolecular disulfide bond, creating an 11-residue cystine loop FP [43], [44]. The ectodomain also contains a 9-residue conserved motif (CM) of unknown function that is invariant in all p10 isolates. The endodomain is the most diverged region of p10, but contains two conserved Cys residues immediately adjacent to the TMD, which in ARV p10 were shown to be palmitoylated [45], followed by a short region of moderately conserved basic residues known as the polybasic (PB) motif (Figure 1); both of these endodomain motifs are required for syncytium formation [45]. To date, relatively little attention has been given to bat reovirus p10 proteins, or to exploiting comparative approaches using the two p10 clades to better define structure-function relationships.

Using ARV strain 176 (hereinafter referred to as ARV) and NBV p10 proteins as representatives of the two clades, we used chimeric p10 constructs to identify the ectodomain as the principal determinant of the differing syncytiogenic efficiencies of these two p10 proteins. Further fine structure mapping revealed the 40-residue ectodomain contains two distinct functional motifs, both of which are essential for syncytiogenesis: an N-terminal ∼25-residue motif governing formation of a cystine loop FP, and a C-terminal 13-residue membrane-proximal ectodomain region (MPER) directing p10 multimerization and clustering into plasma membrane microdomains. Cholesterol-dependent clustering and multimerization are co-dependent, reversible, and species-specific, with the four membrane-adjacent MPER residues dictating homomultimer specificity.

## Results

### The NBV ectodomain confers enhanced cell-cell fusion and lipid-mixing activity to p10

To confirm previous qualitative assessments of ARV and NBV p10 syncytiogenic activity [46], these two representatives of the avian and pteropine p10 lineages were transfected into QM5 fibroblasts and Vero epithelial cells. A quantitative syncytiogenesis assay confirmed NBV p10-induced syncytium formation is markedly faster than ARV p10 in both cell types, with NBV p10 inducing ∼3–4-fold higher levels of cell-cell fusion than ARV p10 by 24 h post-transfection (Figure S1A and B). Western blotting and FACS cell surface analysis using α-FLAG antibodies and FLAG-tagged p10 constructs indicated differing syncytiogenic kinetics were not due to differences in ARV and NBV p10 expression (Figure S1C).

**Figure 2:**
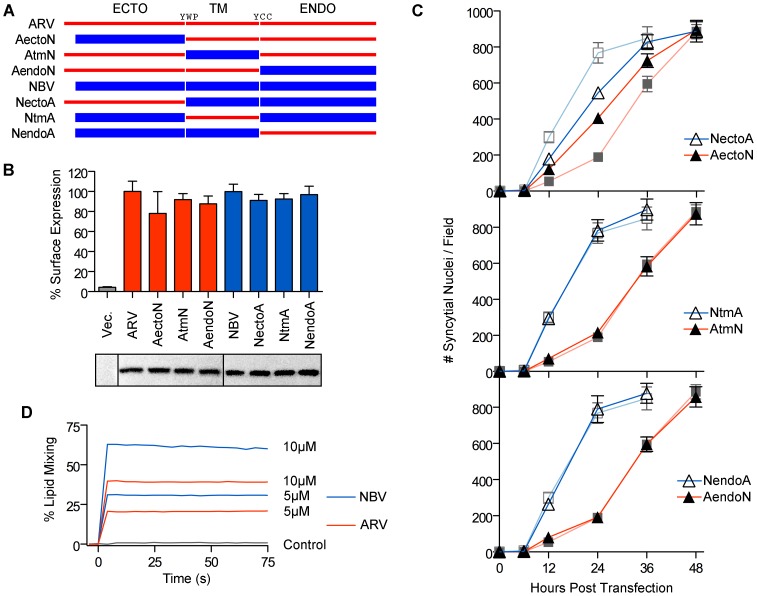
The p10 ectodomains are major determinants of syncytiogenic efficiency and lipid mixing activity. (A) The ecto-, TM and endodomains of ARV (red) and NBV (blue) p10 were permutationally exchanged, using sequential PCR reactions, to create six chimeric p10 constructs named as indicated. (B) Surface expression levels (top) measured by flow cytometry, and total steady-state expression levels (bottom) detected by western blotting, of N-terminally FLAG-tagged versions of the parental p10 and chimeric p10 constructs. Lanes in the western blot were spliced together from a single blot. Surface expression levels are presented as mean ± SEM (n = 3) (C) Syncytial nuclei present in five random fields of Giemsa stained monolayers were counted at indicated times post-transfection for chimeric ecto (top), TM (middle) and endodomain (bottom) p10 constructs in transfected QM5 cell monolayers. Results are mean ± SEM (n = 3). Syncytiogenesis induced by parental ARV and NBV p10 (see Figure S1) are shown in watermark on each graph. (D) Fluorescence resonance energy transfer was used to monitor the extent of lipid mixing induced by 5 µM and 10 µM ARV (red) and NBV (blue) ectodomain synthetic peptides incubated with 100 µM LUVs composed of DOPC-DOPE-cholesterol-sphingomyelin (40∶20∶20∶20). The same assay was performed using buffer instead of peptide (control, black).

We exploited the differing fusogenic activities of the ARV and NBV p10 proteins to define functional motifs that confer enhanced fusion capability. Using sequential PCR reactions, the homologous ectodomains, TMDs and endodomains of ARV and NBV p10 were exchanged to generate six chimeric p10 proteins (Figure 2A). Chimeras were named to reflect the proportion of domains stemming from each parental p10 protein. For example, the chimeric ARV protein containing the NBV ectodomain is termed ARVectoNBV (abbreviated AectoN). FACS analysis and western blotting indicated no significant differences in cell surface and total expression levels of FLAG-tagged versions of the chimeric proteins (Figure 2B). Quantitative syncytiogenesis assays revealed the relative contribution of each domain to the overall syncytiogenic rate (Figure 2C). Exchanging the TMDs or endodomains of ARV and NBV p10 had no effect on the rate or extent of syncytium formation; AtmN and AendoN had identical syncytiogenic kinetics as parental ARV p10 (Figure 2C, bottom two panels). The same situation applied for NBV p10 containing an ARV p10 TMD or endodomain (i.e. NtmA and NendoA chimeras), both of which shared the fusogenic activity of the parental NBV p10 (Figure 2C, bottom two panels). In contrast, an NBV ectodomain substantially increased the syncytiogenic activity of ARV p10 while an ARV ectodomain impaired the fusogenic activity of NBV p10 (Figure 2C, top panel). Interestingly, the two ectodomain chimeras had intermediate syncytiogenic activities relative to the parental p10 proteins, indicating the ectodomains directly contribute to the differing fusion activities of ARV and NBV p10 but require a homotypic TMD or endodomain to confer the full fusion phenotype of the parental proteins.

ARV and NBV p10 ectodomain peptides (40 and 36 residues, respectively) were chemically synthesized to include the intramolecular disulfide bond (confirmed by mass spec analysis), which is required for ARV p10 FP activity [43], and assessed for lipid mixing activity using a commonly employed fluorescent liposome-based assay [44], [47]. Both ectodomain peptides displayed robust, dose-dependent lipid mixing activity, with the NBV peptide inducing approximately twice the extent of lipid mixing as the ARV peptide in a dose-dependent manner (Figure 2D). The ectodomain is therefore the primary determinant of species-specific p10 syncytiogenic efficiency, a property that correlates with relative FP-induced lipid mixing activities.

### The N-terminal p10 ectodomain region contains an essential, species-specific motif required for cystine loop formation

**Figure 3:**
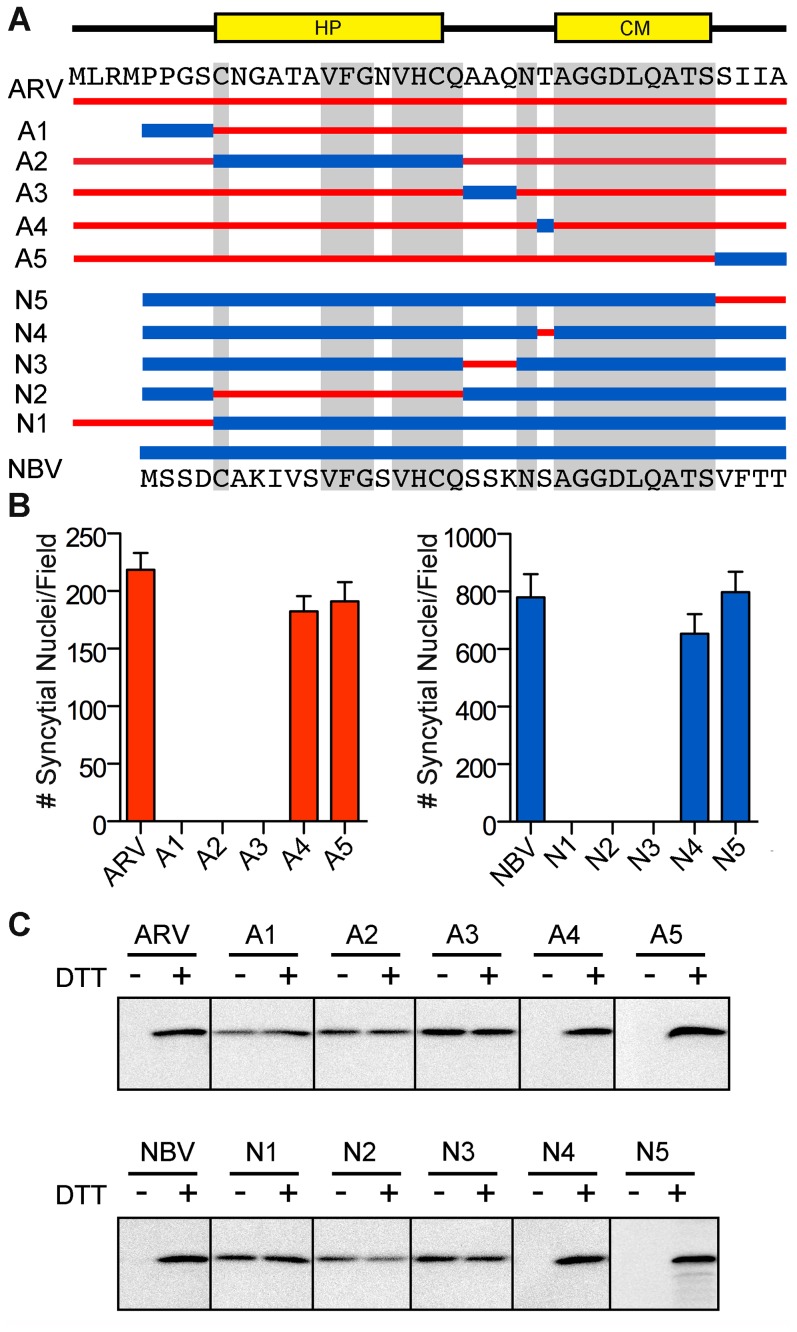
A distinct N-terminal ectodomain motif governs species-specific formation of an essential intramolecular disulfide-bond formation. (A) Ectodomain segments were exchanged between ARV and NBV p10 to create ten chimeric constructs (A/N 1–5). Thin red lines and thick blue lines denote ARV and NBV sequences, respectively. Amino acid sequences of ARV and NBV are shown at the top and bottom, respectively. Background grey boxes denote conserved amino acids. Locations of the HP and CM are indicated above. (B) Syncytium formation induced by each construct in transfected QM5 cells, as determined in Figure 2C, presented as mean ± SEM (n = 3). (C) N-terminally FLAG-tagged versions of the indicated chimeric ectodomain constructs were transfected into QM5 monolayers and at 24 h post-transfection, cells were incubated in HBSS with or without 0.1 mM DTT prior to treatment with membrane-impermeable maleimide-PEG2-biotin to detect free thiol groups in the p10 ectodomain. Biotinylated proteins were isolated using neutravidin beads and detected by western blotting using FLAG-specific antiserum.

To further define motifs within the ectodomain that contribute to fusion potential, six additional constructs were created that exchanged portions of the ARV and NBV p10 ectodomains (Figure 3A). These constructs exchanged sequences bracketing the cystine loop (constructs A1, A3, N1, and N3), the sequences within the Cys-flanked HP (constructs A2 and N2), the non-conserved residue adjacent to the N-terminus of the CM (constructs A4 and N4), and the four membrane-proximal residues (constructs A5 and N5). Quantitative syncytiogenesis assays revealed chimeras composed of residues exchanged within or flanking the HP were all inactive for syncytium formation (Figure 3B). A cell-surface biotinylation assay, previously employed to identify a cysteine loop in ARV p10 [43], determined NBV p10 also forms a cystine loop; cells had to be treated with DTT before plasma-membrane localized NBV p10 could react with the thiol-specific biotinylation reagent (Figure 3C). More notably, fusion-dead ARV and NBV p10 constructs (A1–A3 and N1–N3) could all be biotinylated without DTT treatment, indicating the presence of free thiols and inability to form an intramolecular disulfide bond. Conversely, constructs with substitutions flanking the CM (A4, A5, N4 and N5) remained fusion-active (Figure 3B) and retained the cystine loop (Figure 3C). Thus, the N-terminal ∼25 ectodomain residues of ARV and NBV p10 define a single motif required for species-specific formation and fusion activity of an essential cystine loop-based FP.

### ARV and NBV p10 ectodomains direct homotypic clustering of p10 in cholesterol-dependent plasma membrane microdomains

**Figure 4:**
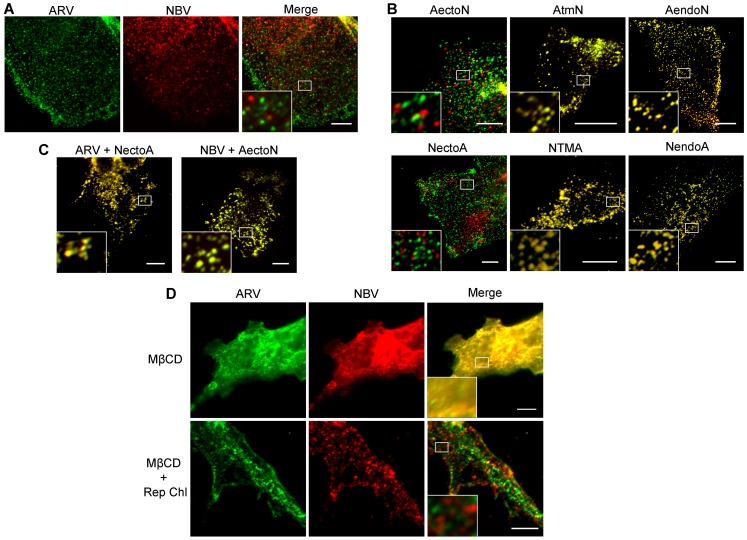
Ectodomain-mediated homotypic clustering of p10 in the plasma membrane is cholesterol-dependent. (A) QM5 cells co-transfected with N-terminally FLAG-tagged ARV p10 and N-terminally myc-tagged NBV p10 were fixed without permeabilization, and surface-localized ARV and NBV p10 detected using mouse-α-FLAG and rabbit-α-myc antisera, respectively. Bound antibodies were detected with Alexa Fluor 488 goat-α-mouse (green) and Alexa Fluor 647 goat-α-rabbit (red), and superposed in the merged image. (B) As in panel A, except cells were co-transfected with N-terminally FLAG-tagged ARV (top row) or NBV (bottom row) p10 and the indicated N-terminally myc-tagged chimeric constructs. (C) As in panel A, except cells were co-transfected with N-terminally FLAG-tagged ARV or NBV p10 and N-terminally myc-tagged versions of the indicated ectodomain chimeras. D) QM5 cell monolayers co-transfected with N-terminally FLAG-tagged ARV p10 and N-terminally myc-tagged NBV p10 were incubated with 20 mM MβCD for 20 min to deplete membrane cholesterol. Cells were then either fixed or cholesterol was repleted by treatment with MβCD-cholesterol complexes for 30 min prior to fixation. Cells were fixed and stained as in panel A. Scale bars = 10 µm. Insets are 400% enlargements of the indicated areas.

QM5 cells were co-transfected with N-terminally FLAG-tagged ARV p10 and N-terminally myc-tagged NBV p10 to allow simultaneous visualization of both proteins on the surface of non-permeabilized cells. Immunofluorescence microscopy revealed both p10 proteins displayed a punctate staining pattern randomly distributed over the cell surface, with little to no colocalization of the ARV and NBV puncta (Figure 4A). In contrast, FLAG- and myc-tagged versions of the same parental protein perfectly colocalized in puncta (Figure S2A), indicating each punctum comprises a homotypic clustering of more than one p10 protein. The previously created chimeric p10 constructs were N-terminally myc-tagged and co-expressed with N-terminally FLAG-tagged parental constructs to define which domain(s) govern segregated surface localization. ARV p10 constructs containing an NBV TMD or endodomain perfectly colocalized with parental ARV p10, while the ARV construct containing the NBV ectodomain displayed almost no colocalization with ARV p10 (Figure 4B, top row). The same situation occurred with the NBV constructs; an ARV ectodomain prevented colocalization with parental NBV p10 while constructs with an ARV TMD or endodomain colocalized with NBV p10 (Figure 4B, bottom row). Colocalization was also observed in cells co-expressing FLAG-tagged ARV p10 and myc-tagged NectoA, and in cells co-expressing FLAG-tagged NBV p10 and myc-tagged AectoN (Figure 4C), indicating p10 ectodomains are both necessary and sufficient to determine homotypic p10 clustering in the plasma membrane.

A similar punctate plasma membrane staining pattern was previously noted for RRV p14 FAST protein, which localizes to cholesterol-rich microdomains that share features with lipid rafts [48]. Acute extraction of cholesterol from QM5 cells expressing N-terminally FLAG-tagged ARV p10 using methyl-β-cyclodextrin (MβCD) resulted in a dose-dependent alteration in p10 plasma membrane staining from punctate to diffuse (Figure S2B). Live-cell fluorescence imaging with C-terminally GFP-tagged p10 indicated the punctate plasma membrane localization of p10 was rapidly converted to a diffuse staining pattern following addition of MβCD to cells (Video S1). Most interestingly, the diffuse, overlapping staining pattern of ARV and NBV p10 in the absence of cholesterol rapidly reverted to segregated, homotypic, punctate staining when membrane cholesterol was restored using cholesterol-loaded MβCD (Figure 4D). Thus, the p10 ectodomain directs the reversible and preferential homotypic clustering of plasma membrane-localized ARV and NBV p10 into species-specific, cholesterol-dependent microdomains.

### Homotypic p10 clustering in the plasma membrane is directed by two MPER motifs

**Figure 5:**
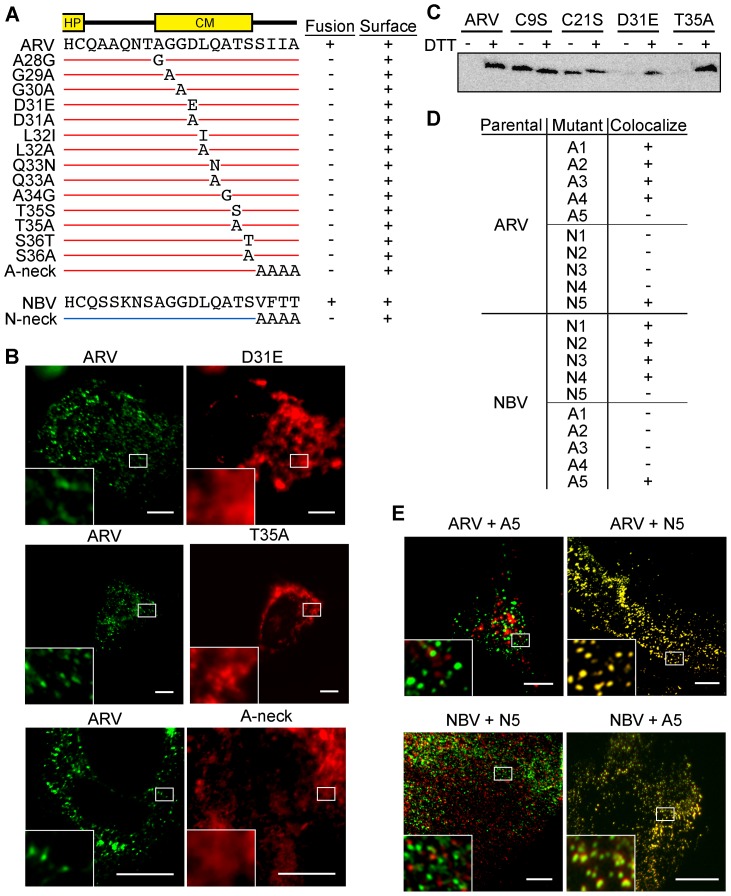
CM and neck region residues are essential for p10 clustering in plasma membranes and neck region residues determine species-specific p10 homotypic clustering. (A) In the context of ARV p10, each non-Ala/Gly CM residue was substituted to either Ala or a conserved amino acid, while each Ala/Gly residue was substituted to the obverse. Constructs are named at left according to the substitution. The tetra-peptide neck residues were substituted to Ala in the context of both ARV (A-neck) and NBV (N-neck) p10. Syncytiogenic activity and cell surface expression levels (+ indicates no significant difference relative to the parental p10 construct) are summarized at right. (B) Representative immunofluorescence images of QM5 cells expressing N-terminally FLAG-tagged ARV p10 or N-terminally myc-tagged ARV p10 containing substitutions in the CM (D31E and T35A), or the A-neck construct, stained as in Figure 4. (C) N-terminally FLAG-tagged versions of the indicated point substitution constructs were subjected to the surface biotinylation assay to detect formation of the intramolecular disulfide bond as in Figure 3C. (D) Summary table of colocalization results obtained from co-transfections of QM5 cells with N-terminally FLAG-tagged versions of ARV or NBV p10 and the indicated N-terminally myc-tagged p10 ectodomain chimeras, listed in Figure 3. (E) Representative images of QM5 cells co-expressing N-terminally FLAG-tagged ARV or NBV p10 and N-terminally myc-tagged chimeric neck constructs (A5 and N5 from Figure 3). Cell surface-localized p10 was detected by immunofluorescence microscopy as in Figure 4, and merged images are shown. For all images, scale bars = 10 µm and insets are 400% enlargements of the indicated areas.

A series of ARV constructs containing point substitutions in the ectodomain were used for fine structure mapping of sequences governing p10 clustering in membrane microdomains. A C9S substitution in ARV p10, which abrogates cystine loop formation and syncytiogenesis [43], had no effect on p10 clustering (Figure S2C), indicating the cystine loop FP motif does not influence p10 cell surface distribution. To determine whether the CM plays any role in p10 microdomain association, each non-Ala/Gly residue of the ARV p10 CM was individually point-substituted with either a conserved residue or with Ala, while each Ala/Gly residue was individually substituted to the obverse (Figure 5A). Surface immunofluorescence and syncytiogenesis assays with these 14 CM point substitutions revealed changes to the CM eliminated p10-induced syncytium formation (Figure 5A) and generated a diffuse surface-staining pattern (Figure 5B, top and middle rows). The four diverged, juxtamembrane residues of the MPER, hereinafter referred to as the neck region, connect the TMD to the CM and can be exchanged in the ARV and NBV p10 proteins with no effects on syncytiogenesis (Figure 3B). However, substitution of these neck residues with Ala had the same effect as CM substitutions, abrogating both p10-induced syncytium formation (Figure 5A) and clustering in membrane microdomains (Figure 5B, bottom row). The p10 MPER, encompassing CM and neck residues, therefore governs the essential clustering of p10 in cholesterol-rich membrane microdomains.

To identify regions responsible for homotypic clustering of ARV and NBV p10, the previously constructed chimeric ectodomain constructs (i.e., those constructs where diverged ectodomain sequences were exchanged) were co-expressed with each of the parental p10 constructs. Surface immunofluorescence microscopy revealed ARV and NBV p10 each colocalized homotypically with chimeric constructs containing heterotypic sequences in or flanking the HP, or on the N-proximal side of the CM (the A/N 1, 2, 3 and 4 constructs) (Figure 5D). In contrast, exchange of the four-residue neck region converted homotypic clustering to heterotypic clustering; NBV p10 perfectly colocalized with ARV p10 containing the tetra-peptide neck residues from NBV, and *visa versa* (Figure 5D and E). Thus, the juxtamembrane tetra-peptide is solely responsible for species-specific homotypic clustering of p10 in cholesterol-dependent plasma membrane microdomains.

### Co-clustering of fusion incompetent p10 constructs with parental p10 exerts a dominant-negative effect on syncytiogenesis

**Figure 6:**
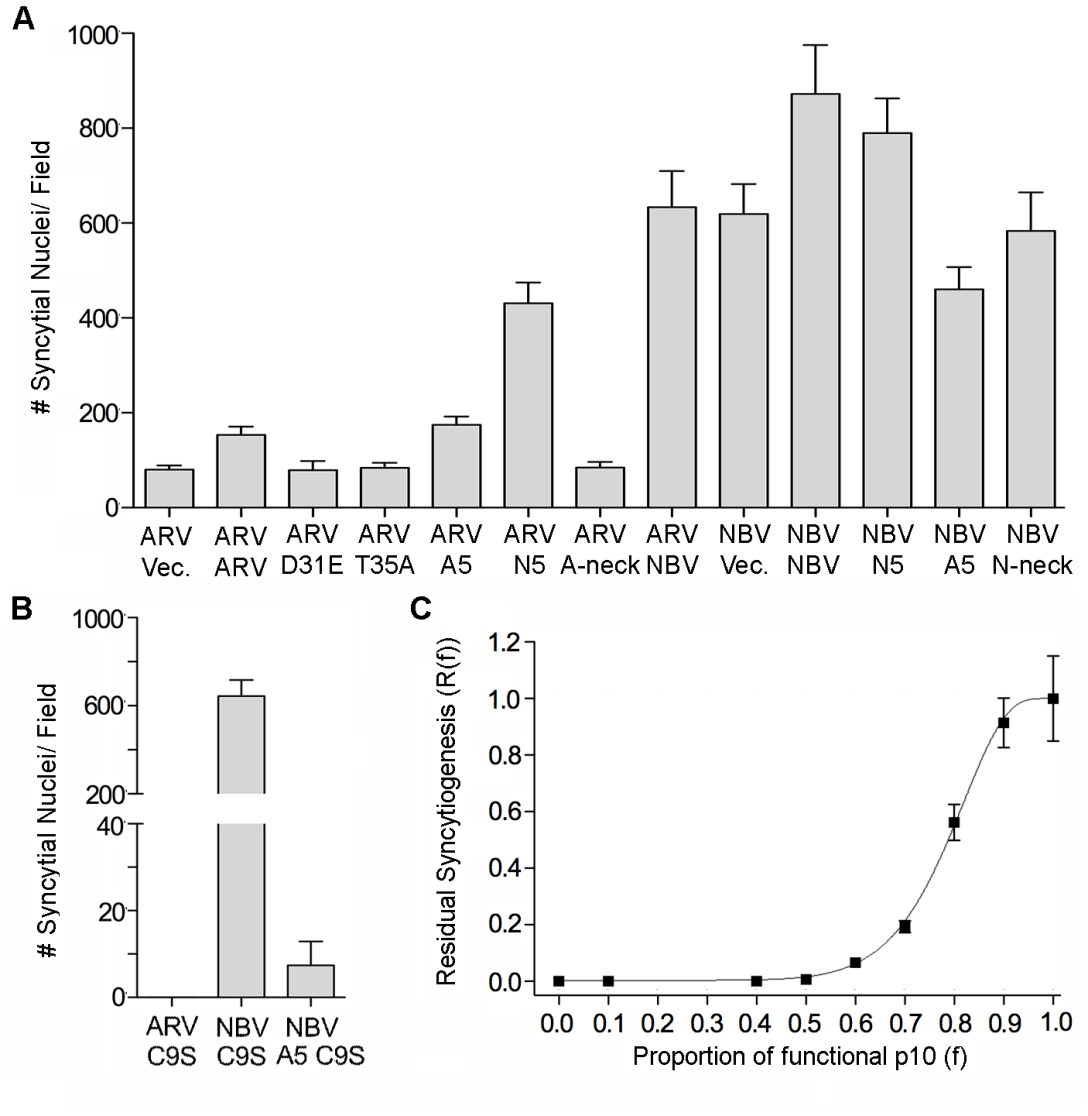
Effects of p10 chimeras and point substitutions on fusion activity of parental p10. (A) Syncytium formation in QM5 cells co-transfected with ARV or NBV p10 and the indicated co-transfectants or empty vector (vec) was quantified at 24 h post-transfection as described in Figure 2C, and results are presented as mean ± SEM (n = 3). Co-transfectants included CM point substitutions (D31E and T35A), chimeric neck constructs (A5 and N5), and Ala substitutions of the tetra-peptide neck residues (A-neck and N-neck), as depicted in Figure 5. (B) As in panel A, except cells were co-transfected with ARV or NBV p10 and the ARV p10 C9S substitution, or with NBV p10 and the C9S substitution in an A5 background. Results are presented as mean ± SEM (n = 3). (C) Co-transfections with varying ratios of functional NBV p10 (*f*) and non-functional ARV C9S with the A5 substitution. Data is presented as mean ± SEM (n = 3).

Neck region exchanges redirected p10 to a heterotypic clustering pattern (Figure 5D and E) but had no effect on disulfide loop formation (Figure 3C) or syncytiogenic activity (Figure 3B), indicating localization of ARV and NBV p10 to discrete membrane microdomains does not contribute to differing fusion efficiencies of these FAST proteins. To further explore the relationship between microdomain association and fusion activity, syncytium formation was assessed in QM5 cells co-transfected with a parental p10 protein and equivalent amounts of plasmid DNA expressing one of several chimeric or substituted constructs that had various effects on p10 clustering and syncytiogenesis. Co-transfecting cells with the same parental p10 (i.e. twice the dose of p10-expressing plasmid DNA) increased syncytiogenesis ∼50–100% relative to cells co-transfected with empty vector (e.g., ARV+ARV relative to ARV+vector, and NBV+NBV relative to NBV+vector; Figure 6A). Co-expressing parental p10 with constructs defective in both syncytium formation and clustering (e.g. CM mutants or Ala substitutions of the neck regions) had the same effect as co-transfecting empty vector; these constructs had no effect on parental p10-induced syncytiogenesis (Figure 6A). Co-expressing the ARV p10 C9S mutant, which is fusion-incompetent but retains the MPER homotypic microdomain association motif, eliminated ARV p10-induced syncytium formation but had no effect on NBV p10 syncytiogenesis (Figure 6B). Conversely, a C9S substitution in an ARV p10 A5 background (i.e., ARV p10 containing the NBV p10 neck region) suppressed NBV p10-induced syncytium formation by ∼99% (Figure 6B). Varying the proportion of functional NBV p10 and non-functional ARV A5 C9S yielded a dose-response curve, with 50% maximal syncytiogenesis occurring at a functional∶non-functional p10 ratio of ∼4∶1 (Figure 6C). Fusion-deficient constructs therefore exert a potent dominant-negative effect, but only when co-localized to the same membrane microdomain as a parental p10 protein. Lastly, co-expressing ARV or NBV p10 with heterotypic neck region substitution constructs that co-localize with each other (i.e., ARV p10 with NBV N5, or NBV p10 with ARV A5 in Figure 5D) resulted in approximately equivalent, intermediate fusion phenotypes; syncytium formation was more extensive than that induced by ARV p10 alone but reduced relative to NBV p10 alone (Figure 6A). Taken together, these results indicate each punctum represents a “fusion unit”, with overall fusion efficiency being dictated by the population of p10 proteins present in individual puncta.

### MPER directs ARV and NBV p10 cholesterol-dependent homomultimerization

**Figure 7:**
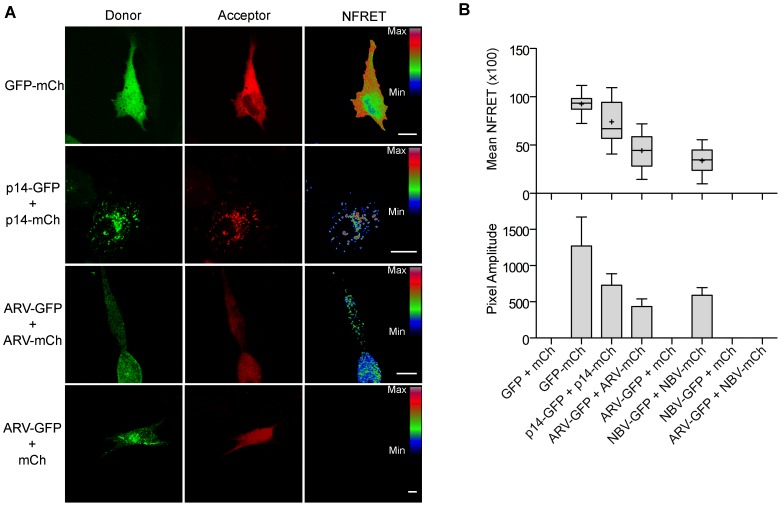
ARV and NBV p10 proteins form homo- but not heteromultimers. (A) Representative images of sensitized emission FRET, showing the donor and acceptor channels, and the calculated normalized FRET (NFRET) image. Control images were obtained from cells expressing EGFP directly linked to mCherry (GFP-mCh) or ARV p10-EGFP cotransfected with free mCherry (ARV-GFP+mCh) as negative FRET controls, or from cells co-expressing p14 FAST proteins linked to EGFP and mCherry as positive FRET controls for a known, multimeric membrane protein. Images acquired from cells co-expressing EGFP- and mCherry-tagged ARV p10 constructs (ARV-GFP+ARV-mCh) were used to detect ARV p10 multimerization. NFRET range is denoted by color gradations. Scale bars = 10 µm. (B) Fitted Gaussian distributions of twenty calculated NFRET images from two separate experiments were used to calculate the mNFRET (top) and mean pixel amplitude (bottom) from cells transfected or co-transfected with the indicated fluorescent probes. Boxes indicate mNFRET standard deviations, + denotes mean mNFRET, lines are the median mNFRET, and whiskers indicate min and max mNFRETs. Error bars in pixel amplitude panel represent standard error propagated within and across experiments.

To determine if puncta contain p10 multimers, we employed fluorescence resonance energy transfer (FRET) *in cellulo*. Since the efficiency of energy transfer between fluorophores varies inversely to the 6^th^ power of the distance between donor and acceptor fluorophores, FRET only occurs if the average spatial separation of the fluorophores is <5–10 nm, a distance only stably observed during direct protein-protein interactions [49]. ARV and NBV p10 proteins were C-terminally tagged with either enhanced green fluorescent protein (EGFP) or a monomeric derivative of red fluorescent protein (mCherry), a FRET pair with good spectral overlap but low donor-acceptor cross-talk levels [50]. The PixFRET Image-J plug-in [51] was used to calculate donor and acceptor spectral bleed-through (SBT) values and normalized FRET (NFRET) intensities using pixel-by-pixel analysis of sensitized emission FRET, and mean NFRET (mNFRET) values were determined for 10 cells in each of two separate experiments. Positive controls included a uni-molecular FRET pair (i.e., EGFP directly attached to mCherry via a flexible linker) and a bi-molecular FRET pair, the multimeric p14 FAST protein, both of which gave positive FRET signals (Figure 7A). The negative controls, ARV-GFP co-expressed with free mCherry (Figure 7B) and ARV-mCherry co-expressed with free GFP (data not shown), yielded no detectible FRET signals.

**Figure 8:**
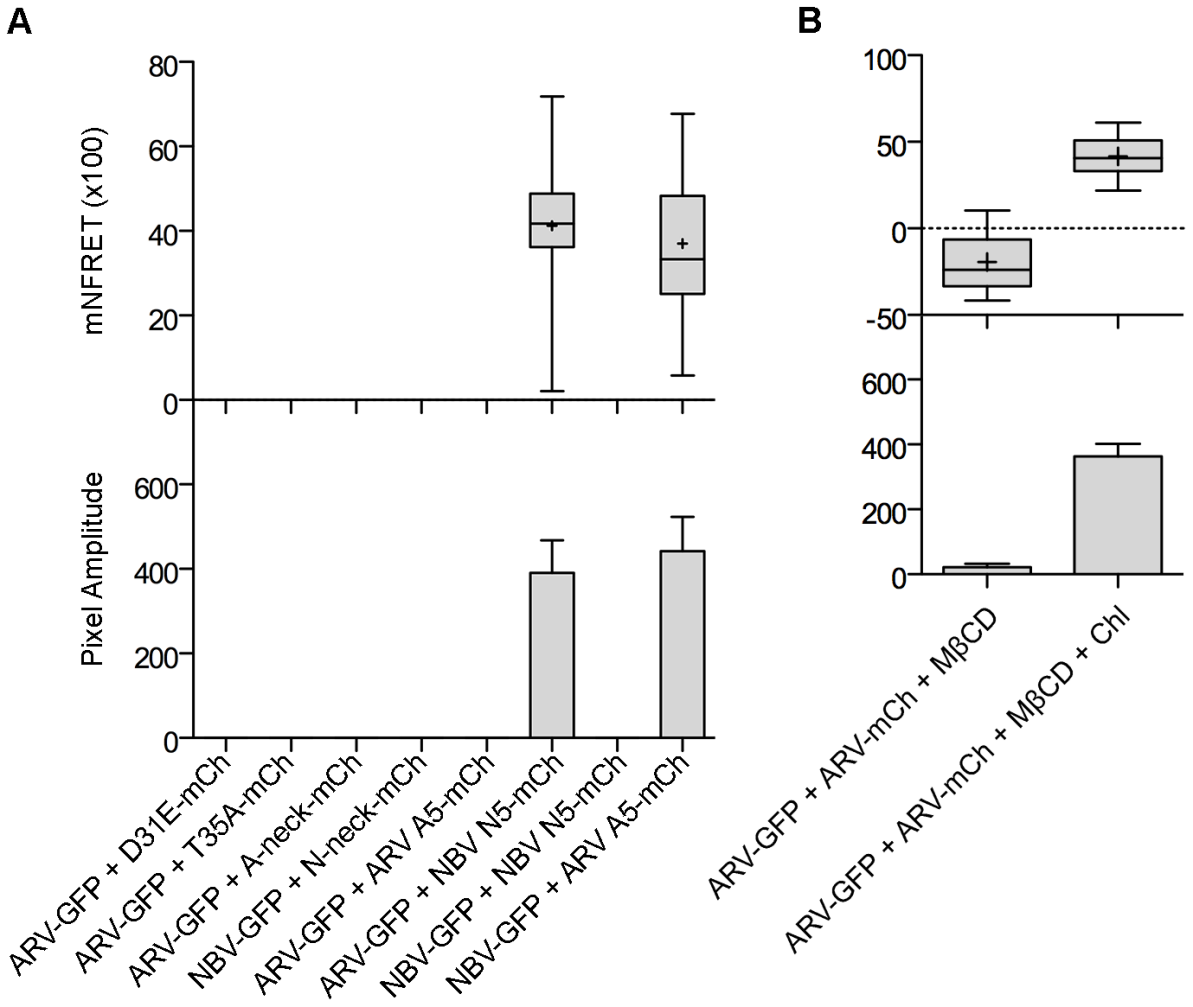
The CM and neck region are both required for p10 multimerization but the neck region alone determines multimer specificity in a cholesterol-dependent manner. (A) Cells co-expressing indicated EGFP and mCherry-tagged constructs with point substitutions in the CM (D31E or T35A), Ala substitutions of the neck tetra-peptide (A-neck or N-neck), and chimeric neck constructs (A5 or N5) were imaged to measure sensitized emission FRET. NFRET values are presented as in Figure 7. (B) Sensitized emission FRET of cells co-expressing EGFP- and mCherry-tagged ARV p10 constructs were imaged after treatment with MβCD. Cells were similarly depleted of cholesterol using MβCD, then repleted using cholesterol-loaded MβCD before imaging. NFRET values are presented as in Figure 7.

As shown in the fluorescence images (Figure 7A) and NFRET quantification (Figure 7B), ARV and NBV p10 both formed homomultimers in cells but failed to heteromultimerize. Two representative CM substitutions that abrogate ARV p10 plasma clustering (D31E and T35A) also eliminated multimerization as shown by loss of FRET signal (Figure 8A). Similarly, co-expressing ARV p10-GFP with mCherry-tagged ARV p10 containing either Ala substitutions of the four neck residues (A-neck construct) or the heterologous neck residues from NBV p10 (ARV A5 construct) eliminated the FRET signal, as did similar co-transfections with NBV p10 constructs (Figure 8A); all of these neck constructs also abrogated fusion activity and p10 clustering in the plasma membrane (Figure 5). Conversely, co-expression of ARV p10 with the NBV N5 construct (NBV p10 with the four neck residues of ARV p10), or co-expression of NBV p10 with the corresponding ARV A5 construct, both generated positive FRET signals (Figure 8A) and co-clustered in the plasma membrane (Figure 5). Additionally, cholesterol extraction with MβCD eliminated the detectible FRET signal (Figure 8B), indicating p10 multimerization and p10 clustering in the plasma membrane are both cholesterol-dependent. The MPER therefore controls cholesterol-dependent, reversible homomultimerization and clustering of p10 in plasma membrane fusion platforms, while the tetra-peptide neck residues are solely responsible for determining multimer specificity.

## Discussion

Reovirus-induced syncytium formation is a correlate of virulence [22], [23], the fusogenic reoviruses evolved from at least two (and possibly three or more) gain-of-function events suggesting additional fusogenic reoviruses might emerge [20], and the fusogenic pteropine orthoreoviruses represent a potential threat as emerging human pathogens [13]. Understanding what dictates the fusogenic capacity of the FAST proteins is therefore of some interest. Our comparative analysis of the homologous avian and pteropine orthoreovirus p10 FAST proteins revealed the small ectodomains of these rudimentary fusogens operate as remarkably compact, multifunctional fusion modules that function as the predominant determinant of species-specific p10 fusion efficiency. Mechanistically, the ectodomain comprises two separate components, one of which directs formation of a cystine loop FP and the other co-dependent clustering and multimerization of p10 in plasma membrane microdomains. Plasma membrane clustering and multimerization are cholesterol-dependent, required for syncytiogenesis, reversible, and governed by the MPER, which includes a juxtamembrane tetra-peptide solely responsible for species-specific p10 clustering in microdomains and homomultimerization. Most notably, co-transfections revealed that overall syncytiogenic efficiency is dictated by the p10 composition of individual microdomains, identifying these p10 plasma membrane clusters as multimeric FAST protein fusion platforms.

Differences in p10 fusion efficiency map primarily to the more conserved ectodomain rather than to the highly diverged endodomains (44% versus 19% sequence identity, respectively). While fusion efficiency correlated with the parental source of the ectodomain, chimeras required a homotypic TMD or endodomain to display the full fusion capacity of the parental p10 protein contributing the ectodomain. A similar situation occurs with influenza virus hemagglutinin (HA), although in this instance it reflects the preference for a homotypic combination of TMD and endodomain [52]. How these individual domains function in concert during the fusion process has not been determined, either for the FAST proteins or for enveloped virus fusogens. However, prior studies determined FAST protein TMDs and endodomains are required for fusion pore formation and expansion [32], [53], [54], while their ectodomains mediate the early lipid mixing stage of membrane fusion [43], [44], [47], [55]. Ectodomain-mediated lipid mixing leading to pore formation may therefore be more efficient when pore stabilization and expansion are mediated by a homotypic TMD and/or endodomain.

The present results also implicate relative lipid mixing activities of the ARV and NBV p10 ectodomain cystine loop FPs in the differing syncytiogenic efficiencies of these FAST proteins. Changing the clustering pattern of p10 by exchanging MPER neck motifs did not alter syncytiogenic activity of the parental p10 constructs. For example, the A5 construct (ARV p10 with an NBV neck) had an NBV plasma membrane segregation phenotype (Figure 5) but an ARV p10 fusion phenotype (Figure 3), indicating fusion efficiency is not affected by the species-specific microdomain with which p10 associates. Lipid mixing activities of the p10 ectodomain peptides did, however, correlate with p10 fusion efficiency (Figure 2D). The ARV p10 ectodomain was recently shown to form an essential cystine loop FP [43], results we have now extended to NBV p10 (Figure 3). Furthermore, the ARV and NBV cystine loops are not interchangeable, a reflection of the remarkable sensitivity of disulfide bond formation to species-specific sequences within, and flanking, the p10 loop (Figure 3). Remarkably, despite the extreme sensitivity of disulfide bond formation to minor sequence changes, substitutions in the CM (D31E and T35A) that disrupt clustering and multimerization did not affect disulfide bond formation (Figures 5 and 8), indicating monomeric p10 contains the essential cystine loop FP and is theoretically functional (see below). The N-terminal 21–25 residues of p10 have therefore evolved as an independent structural and functional motif whose sequence-specific geometry is required for formation or stability of an essential cystine loop FP.

We also identified a second independent ectodomain motif that defines a new role for a viral fusogen MPER, namely cholesterol-dependent homomultimerization. MPERs in viral fusogens tend to be short (∼10–20 residues), hydrophobic or amphiphilic sequences frequently enriched in aromatic residues [42]. Their ability to adopt amphipathic helical structures, self-aggregate, and partition into membrane interfacial regions suggests the role of MPERs in membrane fusion reflects their ability to perturb bilayer structure [38], [39], [41], [56], [57]. In contrast, the 13-residue MPERs of ARV and NBV p10 are mostly polar and almost devoid of aromatic residues, and their essential role in membrane fusion is to direct reversible, cholesterol-dependent p10 multimerization. Although noted as an invariant sequence in all p10 isolates from avian and pteropine hosts, no functions have previously been ascribed to the CM. FRET analysis and a panel of CM point substitutions suggested a striking requirement for sequence-specific, spatial self-complementarity in the CM to generate a binding interface suitable for p10 multimerization. While homotypic CM interactions are necessary for multimerization, they are not sufficient; stable multimerization only occurred in conjunction with cholesterol-dependent clustering of p10 in plasma membrane microdomains. Cholesterol-dependent lateral segregation of proteins and lipids creates submicroscopic membrane domains with unique biophysical properties, such as increased membrane thickness and altered lipid order [58]; these or other features of lipid microdomains appear to be needed to stabilize low-affinity p10 multimer interactions. Similar co-dependent raft assembly and multimerization have been reported in other systems [59]–[61]. While cholesterol microdomains have been implicated in the function of numerous enveloped virus fusogens [62], we are unaware of any instance where they are needed for enveloped virus fusogen multimerization. The one exception is Semliki Forest virus E1, where FP binding to sterol- and sphingolipid-rich microdomains in the target membrane is required for low-pH-dependent conversion of E1 pre-fusion heterodimers to fusion active homotrimers [63]. In contrast, the cholesterol-dependence of p10 multimerization reflects an undefined functional interaction between the MPER and donor membrane cholesterol needed to convert p10 monomers to stable multimers.

Indirect evidence also suggests p10 microdomains contain higher-order p10 complexes and function as fusion platforms. The ARV p10 construct containing the neck region of NBV (A5 construct) co-clusters with NBV p10 (Figure 5), while an ARV p10 C9S substitution prevents formation of the cystine loop and eliminates cell-cell fusion (Figure 5) but has no effect on p10 homotypic clustering (Figure S2C). Co-expression of NBV p10 with an equimolar amount of an A5 construct containing the C9S substitution resulted in a 99.15% decrease in NBV p10 syncytiogenesis (Figure 6B). Assuming incorporation of one non-functional p10 polypeptide into a punctum is sufficient to eliminate fusion activity of the complex, then the 0.85±0.53% (SEM, n = 3) of retained syncytiogenic activity would be attributable to clusters containing only parental NBV p10. Based on a binomial distribution, the probability of this percent of clusters containing just parental NBV p10 polypeptides is best approximated by heptameric or octameric clustering (probability of 0.78% and 0.39%, respectively). This interpretation assumes there is a single fusion site formed per cell. It is likely that several functional “fusion platforms” are formed on a single cell. By varying the proportion of functional NBV p10 and non-functional ARV A5 C9S (Figure 6C), we were able to consider the influence of multiple fusion sites on our previous analysis, as previously reported for rabies virus G protein [64]. Residual fusion activity should equal 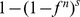 (equation 1), where *f* is the proportion of functional p10 proteins, *n* is the number of p10 proteins in a cluster, and *s* is the number of fusion sites. Fitting our titration data (Figure 6C) to this equation returned best-fit values of *n* = 8.74±0.70 and *s* = 5.17±0.94. We interpret these estimates as an indicator that clusters comprise higher-order assemblies of p10 monomers in the range of octamers.

Cholesterol-dependent, higher-order multimerization is evident with other proteins, such as HIV-1 Gag, where initial membrane binding by lower-order (dimers) Gag multimers progresses to higher-order Gag-Gag interactions in lipid rafts [65]–[67]. The clear correlation between p10 clustering and multimerization in membrane microdomains with p10 fusion competency also suggests microdomains increase local plasma membrane concentrations of p10 to generate fusion platforms, similar to cholesterol-dependent assembly of exocytic SNARE complexes into fusion sites [68]. This conclusion is strengthened by results indicating only non-functional constructs that co-clustered and co-multimerized with parental p10 proteins exerted dominant-negative effects. For example, CM substitutions (D31E and T35A) display a diffuse plasma membrane staining pattern (Figure 5B), do not multimerize with ARV p10 (Figure 8) and exert no dominant-negative effects (Figure 6A). Conversely, fusion dead constructs that colocalized with parental p10 (e.g. ARV p10 C9S) inhibited parental fusion activity (Figure 6B), while functional neck region chimeras generated heterotypic puncta and a syncytiogenic phenotype intermediate between ARV and NBV p10 proteins (Figure 6A). Overall syncytiogenic efficiency is therefore determined not by the number of p10 microdomains but by their compositional p10 bias, indicating individual puncta define a fusion unit.

While the CM and neck region of the MPER both control microdomain association and multimerization, the tetra-peptide neck is solely responsible for segregation of p10 into species-specific microdomains. How proteins partition into lipid rafts remains an unresolved issue in membrane biology [58]. Recent developments in the field suggest the presence of compositionally distinct membrane microdomains differing in their lipid composition, length and asymmetry of N-acyl fatty acids, and hydration status of polar headgroups. These biophysical properties provide lipid environments capable of discriminating between different membrane-associated proteins [69]. For example, HIV env and Ebola GP segregate to different lipid microdomains, both of which can be recruited by Gag to generate HIV particles with a single type of glycoprotein [70]. Proteins may also directly contribute to raft heterogeneity by specifically recruiting and stabilizing a localized lipid environment. Palmitoylation of membrane-proximal cysteines and hydrophobic matching between the lengths of acyl chains and TMDs are the two most common protein features associated with raft recruitment and protein localization to membrane microdomains [71], [72]. As we now show, MPERs can also function as microdomain sorting signals. The tetra-peptide neck of p10 provides a remarkably specific sorting signal capable of segregating two homologous membrane proteins into distinct cholesterol-dependent microdomains. This sorting event occurs in the plasma membrane, and is fully reversible following cholesterol depletion and repletion.

There are several feasible explanations for how the p10 neck might provide both microdomain and multimerization specificity. In a protein-centric-model, the neck would function as the specificity determinant of the MPER multimerization motif, directing homotypic lower-order multimerization. Lipid recruitment by these p10 nanoclusters to generate a sterically favored lipid environment would then recruit additional monomers or lower-order multimers to create a fusion platform. In a lipid-centric model, the tetra-peptide p10 sorting signal functions primarily as a lipid recognition or adaptation motif. Neck residues could interact with specific lipid headgroups in the exoplasmic leaflet, similar to the reported cholesterol-binding activity of the conserved LWYIK juxtamembrane peptide of HIV gp41 [73]. Alternately, the neck may affect hydrophobic matching of the p10 TMD to acyl chain lengths of adjacent lipids. Recent molecular dynamic simulations and studies in artificial bilayers indicate cholesterol decreases the effects of hydrophobic mismatch by altering bilayer thickness and lipid packing, and by inducing changes in TMD helix length [71]. We note that the ARV and NBV p10 necks display very different arrangements of polar and non-polar residues (Figure 1); ARV p10 has a polar-apolar-TMD arrangement while NBV p10 is apolar-polar-TMD. These different arrangements may influence cholesterol-mediated hydrophobic matching and lateral segregation of ARV and NBV p10 into distinct lipid microenvironments. The co-dependent and inseparable relationship between p10 multimerization and microdomain clustering makes it unclear, in either protein- or lipid-centric models, whether p10 associates with, or assembles, lipid rafts to promote multimerization.

Lastly, the readily reversible nature of p10 multimerization in the plasma membrane was an intriguing, and potentially functionally significant, observation. In contrast to the functionally reversible nature of p10 multimerization, enveloped fusogens are locked in metastable dimeric or trimeric pre-fusion conformations that generally undergo an irreversible conformational conversion to a post-fusion, stable trimeric conformation, releasing energy to drive the fusion reaction [34], [36]. Thermodynamically favorable, reversible p10 multimerization implies fusion is not dependent on such energy releasing conformational changes. Furthermore, the co-dependence of multimerization and microdomain association suggests p10 may be capable of shuttling in and out of lipid rafts during the fusion reaction, similar to what occurs with the fusion machinery during peroxisome fusion [74]. Taken together with results indicating monomeric p10 still contains the essential cystine noose FP, this suggests there may be a role for reversible p10 multimerization during the fusion process. Interestingly, conformational changes of enveloped virus fusogens during transition from the pre-fusion structure to the post-fusion, trimeric hairpin conformation are impossible unless the ternary structure is dissociated [75]. Recent studies suggest that monomeric intermediates exist for both tick-borne encephalitis (TBE) virus E [76] and VSV G fusion proteins [77]. Monomeric intermediates and dynamic clustering and dispersion of protein fusogens at sites of fusion, either raft-mediated or otherwise, may therefore be a functionally relevant feature of viral fusogens.

## Materials and Methods

### Cells and reagents

QM5 and Vero cells were grown and maintained as previously described (Corcoran and Duncan, 2004). Rabbit antisera generated against full-length ARV p10 or ARV p10 endodomain were previously described [43], [78]. Monoclonal mouse anti-FLAG (Sigma-Aldrich) and rabbit anti-c-myc (Sigma-Aldrich) antibodies, horseradish peroxidase (HRP)-conjugated goat anti-rabbit (Santa Cruz) and goat-anti mouse (Santa Cruz) antibodies, Alexa Fluor 488-conjugated goat anti-mouse (Invitrogen) and goat anti-rabbit (Invitrogen), antibodies, Alexa Fluor 647-conjugated goat anti-rabbit IgG (Invitrogen), maleimide-PEG2-biotin (Thermo Scientific), neutravidin agarose resin (Thermo Scientific), and methyl-β-cyclodextrin (Sigma-Aldrich) were purchased from the indicated commercial sources.

### Plasmids and cloning

ARV p10 and NBV p10 subcloned into pcDNA3 mammalian expression vectors were previously described [79]. Full domain exchanges and ectodomain segment exchanges between ARV and NBV p10 were created using sequential PCR reactions with custom oligonucleotide primers and clones into pcDNA3. The QuickChange site-directed mutagenesis kit (Agilent Technologies) was used according to the manufacturer's instructions to generate point substitutions for all CM and neck region constructs. A triple FLAG tag was added to the N-terminus of indicated p10 constructs by PCR amplification and cloning. Custom oligonucleotide primers were purchased from IDT, and all constructs were confirmed by sequencing.

### Transfections and syncytial indexing

QM5 or Vero cells were transfected using Polyethylimine (PEI, Polysciences Inc.) as per manufacturer's instructions. Syncytial indexing was performed on 50% confluent monolayers in 12-well plates transfected with 0.5 µg of plasmid DNA (unless otherwise stated). At 4 h post- transfection, the transfection mix was replaced with Earle's 199 growth media (Gibco) supplemented with 10% fetal bovine serum (Sigma-Aldrich). At indicated times post-transfection, cells were fixed with methanol and Wright-Giemsa stained (Siemens Healthcare Diagnostics). Stained monolayers were imaged using a Nikon DIAPHOT-TMD under 200× magnification. The numbers of syncytial nuclei were counted from five random fields per well, in three separate experiments, and the syncytial index reported as the mean ± SEM propagating errors within and across experiments. Data in Figure 6C was fit to the following equation, described in Roche and Godin [64], using the curve fitting toolbox 3.3 application in MATLAB R2012b:
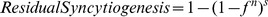
(1)where *f* is the proportion of functional p10 proteins, *n* is the number of p10 proteins in a cluster, and *s* is the number of fusion sites.

### SDS-PAGE and western blotting

SDS-PAGE and Western blotting were carried out as previously described [43], [53]. A 1∶10,000 dilution of anti-FLAG mouse antiserum followed by a 1∶10,000 dilution of HRP-conjugated goat anti-mouse secondary antibody was used to measure overall protein expression levels. Western blots were developed using ECLplus western blotting reagent (GE Healthcare) and imaged on a Kodak 4000 mm Pro CCD imager.

### Surface expression by flow cytometry

Transfected QM5 monolayers were incubated for 24 h in Earle's 199 growth media supplemented with 10% fetal bovine serum. Live cells were immunolabeled at 4°C with 1∶200 dilution of mouse anti-FLAG primary antibody, followed by 1∶2000 dilution of Alexa 647-conjugated goat anti-mouse antibody, as previously described [43], [53]. Cells were resuspended with 50 mM EDTA in PBS, fixed in 3.7% formaldehyde, and surface expression was quantified using a FACSCalibur flow cytometer (Becton Dickinson) by counting 20,000 cells. Cell surface fluorescence was analyzed using FCS Express 2.0 (De Novo Software).

### Cell surface biotinyation assay

The presence on an intramolecular disulfide bond in the ectodomain of FLAG-tagged p10 constructs was detected as previously described [43]. QM5 cells seeded in 10 cm dishes (Corning) at ∼50% confluency were transfected with the indicated constructs. At 24 h post-transfection cells were washed twice with Hanks buffered saline solution (HBSS) before being incubated in HBSS with or without 0.1 mM dithiothreitol (DTT) for five min. Cells were washed three times with HBSS, then incubated with shaking in 1 µg/ml maliemide-PEG_2_-biotin (a membrane impermeable biotinylation reagent) for 25 min at 4°C to biotinylate free thiol groups on the cell surface. Cells were washed four times with HBSS to remove excess biotin reagent, then once with HBSS containing 1% BSA to quench residual biotinylation reagent. Cells were washed twice with PBS, then resuspended with 50 mM EDTA in PBS, lysed in RIPA buffer (Tris, pH 8.0, 150 mM NaCl, 1 mM EDTA, 1% NP40, 0.5% NaDOC) with protease inhibitors (Pierce), the lysate was incubated overnight with neutravidin agarose resin to pull down biotinylated proteins, and pellets were boiled in protein sample buffer with 100 mM DTT to release biotinylated proteins. Samples were then analyzed via SDS-PAGE and Western blotting.

### Cell surface immunofluorescence microscopy

QM5 cells grown on coverslips at approximately 50% confluence were transfected with FLAG- or c-myc-tagged versions of ARV or NBV p10 constructs as described above. At 24 h post-transfection, cells were fixed with paraformaldehyde, blocked with 1% BSA in PBS for 30 min, and then incubated with 1∶1000 dilutions of mouse anti-FLAG and rabbit anti-c-myc monoclonal antibodies for 1 h at room temperature. After thorough washing with PBS, the cells were incubated with 1∶1000 dilutions of Alexa 488-conjugated goat anti-mouse antibody and Alexa 647-conjugated goat anti-rabbit antibody for 1 h at room temperature. Coverslips were then mounted and sealed on slides using prolong gold anti-fade reagent (Invitrogen), then imaged using an Axiovert 200M inverted microscope (Zeiss).

### Lipid mixing assay

A fluorescence resonance energy transfer (FRET)-based mixing assay was employed to monitor the lipid-mixing potential of synthetic p10 ectodomain peptides, as previously described [47]. Large unilamellar vesicles composed of a 40∶20∶20∶20 ratio of 1,2-dioleoyl-*sn*-glycero-3- phosphocholine (DOPC), 1,2-dioleoyl-*sn*-glycero- 3-phosphoethanolamine (DOPE), cholesterol and sphingomylein (Avanti Polar Lipids) in 10 mM phosphate buffer with 100 mM NaF, pH 7.4, were prepared by extrusion through 100 nm polycarbonate filter. A fluorescently-labeled liposome population was similarly prepared to contain 2 mol% each of 1,2-dioleoyl-*sn*-glycero-3-phosphoethanolamine-N-(7-nitro-2-1,3- benzoxadiazol-4-yl) (NBD-DOPE) and 1,2- dioleoyl-*sn*-glycero-3-phosphoethanolamine-N-4(lissamine rhodamine B sulfonyl) (Rho-DOPE). Non-labelled and labeled liposomes were incubated at 37°C in a 9∶1 ratio at a concentration of 100 mM prior to addition of ARV or NBV p10 synthetic peptides dissolved in the same buffer as liposomes and added to the indicated final concentrations, or a buffer-only control. Fluorescence was recorded for up to ten minutes. The percent lipid mixing was calculated using equation (2):
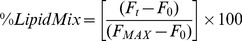
(2)A third liposome population of 0.2 mol% each of NBD-DOPE and Rho-DOPE represented the theoretical maximum (F_MAX_) level of lipid mixing. All experiments were performed in triplicate.

### MβCD cholesterol depletion and repletion

QM5 cell monolayers grown on glass coverslips were transfected with EGFP-tagged NBV p10. At 24 h post-transfection, live cells were imaged using a spinning-disc confocal microscope (3i Intelligent Imaging Innovations, Denver, CO) consisting of a Cell Observer Z1 microscope (Zeiss), an Evolve EMCCD camera (Photometric), and a CSU-X1 spinning disk head (Yokagawa). Images were captured at 1 min intervals to monitor the movement of fluorescently-tagged proteins. At t = 100 sec, 20 mM MβCD in HBSS was circulated into the cell chamber to chelate cholesterol. The diffusion of p10 proteins was followed for an additional 800 sec. Images were acquired using the Slidebook imaging software (Version 5.0). Additionally, QM5 cell monolayers transfected with FLAG-tagged ARV p10 were treated with 2, 5, 10 or 20 mM MβCD for 20 min, then fixed with 3.7% paraformaldehyde. Immunofluorescence staining for surface-localized proteins was performed as described above. Cholesterol repletion experiments were performed as previously described [48] on QM5 cell monolayers transfected with FLAG-tagged ARV p10 and c-myc-tagged NBV p10. Cholesterol-loaded MβCD was made by dissolving cholesterol (6 mg/ml) in serum-free medium containing 20 mM MβCD by vigorous vortexing and heating at 37°C for 30 min, then filtering to remove insoluble cholesterol. Immunofluorescence staining for surface-localized proteins was performed as described above.

### FRET-based multimerization assay

QM5 cells grown on glass coverslips at approximately 50% confluence were transfected with EGFP- and/or mCherry-tagged versions of indicated ARV and NBV p10 mutant constructs. A Zeiss Laser Scanning Microscope (LSM) 510 equipped with a META detector was used in wide field mode to detect sensitized emission FRET. Cells were imaged under a 100× oil immersion, 1.4 NA Plan Apochromat objective. EGFP was excited using a 40 mW Argon laser at 488 nm, and mCherry was excited using a HeNe 548 nm laser. For microscope set-up and control experiments, additional transfections of free EGFP alone, free mCherry alone, free EGFP and free mCherry together, and EGFP directly linked to mCherry (EGFP-mCherry) were done concurrently. The donor and acceptor spectral bleed-through (SBT) values were visually minimized using the free EGFP and free mCherry samples, respectively, prior to detecting FRET in sample conditions. Donor and acceptor SBT ratios were modeled using exponential relationships with fluorophore intensity after exclusion of aberrant background values at low intensities and the application of a Gaussian blur. With the SBT values determined, the normalized FRET (NFRET) for the p10-p10 interaction was calculated. A series of three images was acquired for each cell imaged: (1) sensitized emission FRET image (donor excitation, acceptor emission; (2) donor image (donor excitation, donor emission); and (3) acceptor image (acceptor excitation, acceptor emission). Background subtraction and Gaussian blur of the donor, acceptor and FRET channels were performed on each image stack prior to analysis. Ten images were acquired for each sample condition in duplicate (total of twenty images). Using the PixFRET Image J plugin [51], the FRET intensity of each pixel was normalized to the donor and acceptor expression levels by dividing the FRET-channel pixel intensity by the square-root of the product of the corresponding donor- and acceptor-channel pixels using equation (3):
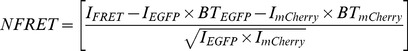
(3)This normalization provided a measure of sensitized emission FRET that is comparable between different samples [80]. Pixel amplitude distributions of the 8-bit NFRET images generated by the PixFRET software were summarized as histograms with a bin width of 0.03906 NFRET units. Each histogram was fit with four Gaussian distributions, and that with the highest calculated R^2^ value was used for further analysis. The mean NFRET (mNFRET) was determined for each image from the best-fit Gaussian distribution. The Gaussian-fitted NFRET histograms were also used to calculate the average pixel amplitude from each condition.

### Accession numbers

GenBank accession numbers for avian and pteropine FAST proteins analyzed in this study. ARV isolates: 176, AAF45151; S1133, AAK18186; Muscovy duck, ABA33820; 138, AAF45154; RAM-1, AAA57266; NC/SEP-R108/03, ABN46970; NC/98, ABL96273; NC/SEP-R61/03, ABN46972; TX99, ABN46974; NC/PEMS/85, ABN46971; Psittacine, ABY78878. Pteropine isolates: Melaka, YP_007507326; Sikamat, AES12474; Pulau, AAR13231; Kampar, ACC77635; NBV, AAF45157.

## Supporting Information

Figure S1 NBV p10 is an inherently better fusogen than ARV p10. (A) Representative images of Geimsa stained QM5 monolayers expressing ARV and NBV p10 at 24 h post-transfection. (B) Syncytiogenesis of ARV (red) and NBV (blue) in transfected QM5 (top) and Vero (bottom) cell monolayers. Syncytial nuclei present in five random fields of Giemsa stained monolayers were counted at indicated times post-transfection and presented as mean ± SEM (n = 3). (C) Surface expression levels (top) measured by flow cytometry, and total steady-state expression levels (bottom) detected by western blotting, of N-terminally FLAG-tagged ARV and NBV p10. Lanes in the western blot were spliced together from a single blot. Surface expression levels are presented as mean ± SEM (n = 3).
(TIF)

Figure S2 (A) ARV and NBV p10 homomultimerize. QM5 cells cotransfected with N-terminally FLAG-tagged and N-terminally myc-tagged ARV or NBV p10 were fixed with paraformaldehyde and surface-localized p10 was detected using mouse-α-FLAG and rabbit-α-myc antisera. Bound antibodies were detected with Alexa Fluor 488 goat-α-mouse (green) and Alexa Fluor 647 goat-α-rabbit (red). Images are merged to show colocalization (yellow). (B) Cholesterol depletion disrupts punctate p10 staining in plasma membranes. QM5 cells co-transfected with N-terminally FLAG-tagged ARV p10 were incubated with the indicated concetrnations of MβCD for 20 min to deplete membrane cholesterol. Cells were then fixed and cell surface-localized p10 was detected as in panel A. (C) Disrupting formation of the ectodomain cystine loop does not affect p10 co-clustering. QM5 cells transfected with N-terminally FLAG-tagged ARV p10 or myc-tagged ARV C9S were fixed and surface-localized p10 was detected as in panel A. Scale bars = 10 µm. Insets are 400% enlargements of the indicated areas.
(TIF)

Video S1 QM5 cells transfected with EGFP-tagged p10 were imaged using a spinning-disc confocal microscope at 100× magnification. At t = 100 sec, 20 mM MβCD was percolated into the sample chamber to chelate membrane cholesterol. Distribution of p10 was followed for an additional 800 seconds. Cells were imaged in the plane of the plasma membrane.
(AVI)
