# Supplementary figures and images for: A Compact, Multifunctional Fusion Module Directs Cholesterol-Dependent Homomultimerization and Syncytiogenic Efficiency of Reovirus p10 FAST Proteins
Source: PLoS Pathog. 2014 Mar 20;10(3):e1004023. doi: 10.1371/journal.ppat.1004023 (PMC3961370; doi:10.1371/journal.ppat.1004023)

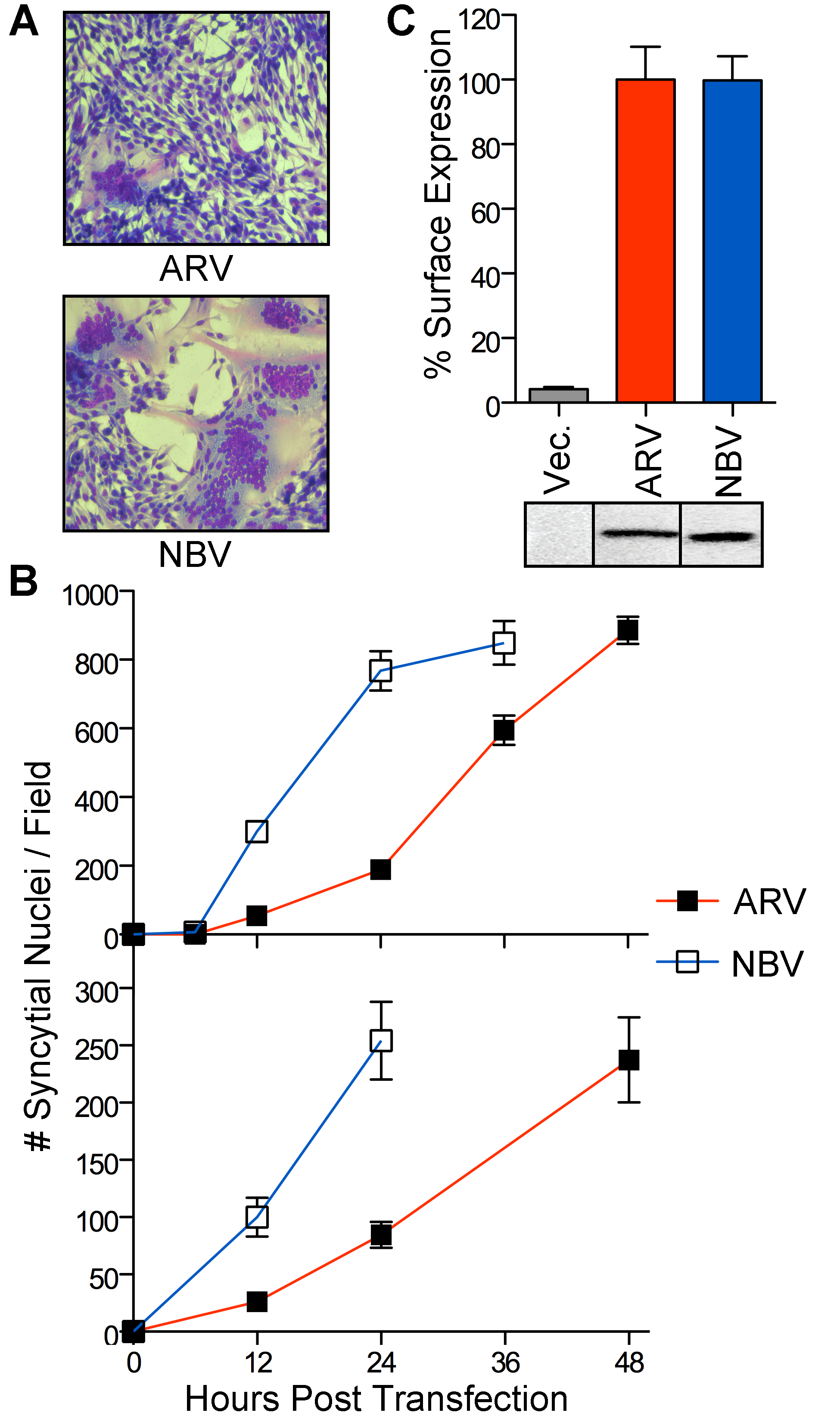

Supplement: Figure S1 — NBV p10 is an inherently better fusogen than ARV p10. (A) Representative images of Geimsa stained QM5 monolayers expressing ARV and NBV p10 at 24 h post-transfection. (B) Syncytiogenesis of ARV (red) and NBV (blue) in transfected QM5 (top) and Vero (bottom) cell monolayers. Syncytial nuclei present in five random fields of Giemsa stained monolayers were counted at indicated times post-transfection and presented as mean ± SEM (n = 3). (C) Surface expression levels (top) measured by flow cytometry, and total steady-state expression levels (bottom) detected by western blotting, of N-terminally FLAG-tagged ARV and NBV p10. Lanes in the western blot were spliced together from a single blot. Surface expression levels are presented as mean ± SEM (n = 3). (TIF) [file ppat.1004023.s001.tif]

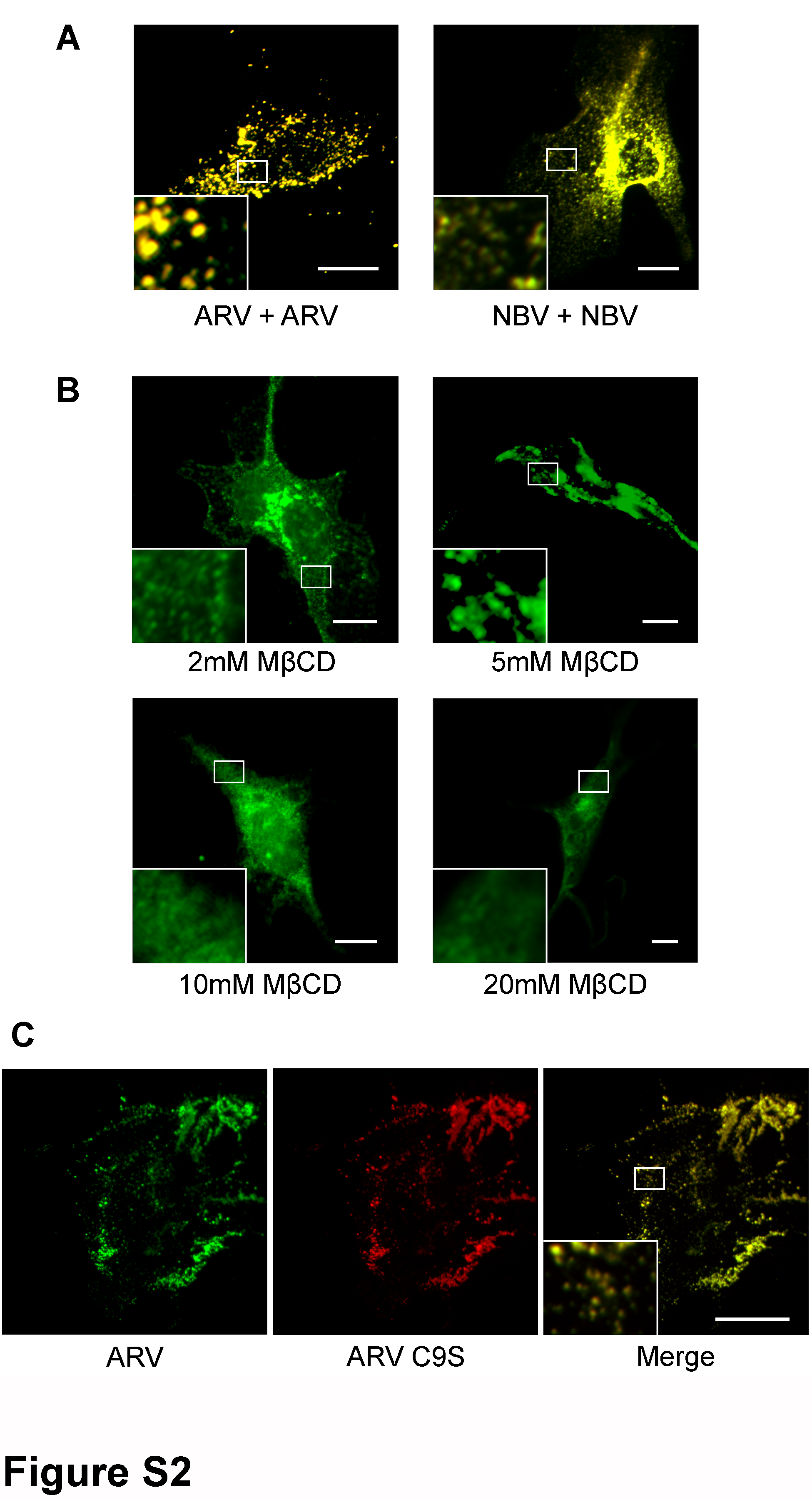

Supplement: Figure S2 — (A) ARV and NBV p10 homomultimerize. QM5 cells cotransfected with N-terminally FLAG-tagged and N-terminally myc-tagged ARV or NBV p10 were fixed with paraformaldehyde and surface-localized p10 was detected using mouse-α-FLAG and rabbit-α-myc antisera. Bound antibodies were detected with Alexa Fluor 488 goat-α-mouse (green) and Alexa Fluor 647 goat-α-rabbit (red). Images are merged to show colocalization (yellow). (B) Cholesterol depletion disrupts punctate p10 staining in plasma membranes. QM5 cells co-transfected with N-terminally FLAG-tagged ARV p10 were incubated with the indicated concetrnations of MβCD for 20 min to deplete membrane cholesterol. Cells were then fixed and cell surface-localized p10 was detected as in panel A. (C) Disrupting formation of the ectodomain cystine loop does not affect p10 co-clustering. QM5 cells transfected with N-terminally FLAG-tagged ARV p10 or myc-tagged ARV C9S were fixed and surface-localized p10 was detected as in panel A. Scale bars = 10 µm. Insets are 400% enlargements of the indicated areas. (TIF) [file ppat.1004023.s002.tif]
